# Census of Hallucinations

**Published:** 1890-10

**Authors:** 


### CENSUS OF HALLUCINATIONS.

Professor William James, Harvard University, Cambridge, Mass., has been appointed to superintend the *Census of Hallucinations*, which was begun several years ago by the "Society for Psychical Research," and of which the International Congress of Experimental Psychology, at Paris, last Summer, assumed the future responsibility, naming a committee in each country to carry on the work.

The object of the inquiry is twofold: First, to get a mass of facts about hallucinations which may serve as a basis for a scientific study of these phenomena; and second, to ascertain approximately the *proportion of persons* who have had such experiences. Until the average frequency of hallucinations in the community is known, it can never be decided whether the so-called "veridical" hallucinations (visions or other "warnings" of the death, etc., of people at a distance) which are so frequently reported, are accidental coincidences or something more.

Some 8,000 or more persons in England, France, and the United States have already returned answers to the questions which heads the census sheets, and which runs as follows:

"*Have you ever, when completely awake, had a vivid impression of seeing or being touched by a living being or inanimate object, or of hearing a voice; which impression, so far as you could discover, was not due to any external physical cause?*"

Any person who has ever had such an impression is requested to communicate the fact to Professor James, at the above address.

It is important that negative answers should be sent as well as the affirmative, for, otherwise, the report will have no value whatever as a census giving percentage of persons having such hallucinations.

